# Interaction mechanisms and kinetics of ferrous ion and hexagonal birnessite in aqueous systems

**DOI:** 10.1186/s12932-015-0031-3

**Published:** 2015-09-22

**Authors:** Tianyu Gao, Yougang Shen, Zhaoheng Jia, Guohong Qiu, Fan Liu, Yashan Zhang, Xionghan Feng, Chongfa Cai

**Affiliations:** Key Laboratory of Arable Land Conservation (Middle and Lower Reaches of Yangtse River), Ministry of Agriculture, College of Resources and Environment, Huazhong Agricultural University, Wuhan, 430070 People’s Republic of China; Department of Chemistry, University of Connecticut, Storrs, 55 North Eagleville Road, Storrs, CT 06269 USA

**Keywords:** Birnessite, Ferrous ion, Redox, Transformation, Lepidocrocite, Goethite

## Abstract

**Background:**

In soils and sediments, manganese oxides and oxygen usually participate in the oxidation of ferrous ions. There is limited information concerning the interaction process and mechanisms of ferrous ions and manganese oxides. The influence of air (oxygen) on reaction process and kinetics has been seldom studied. Because redox reactions usually occur in open systems, the participation of air needs to be further investigated.

**Results:**

To simulate this process, hexagonal birnessite was prepared and used to oxidize ferrous ions in anoxic and aerobic aqueous systems. The influence of pH, concentration, temperature, and presence of air (oxygen) on the redox rate was studied. The redox reaction of birnessite and ferrous ions was accompanied by the release of Mn^2+^ and K^+^ ions, a significant decrease in Fe^2+^ concentration, and the formation of mixed lepidocrocite and goethite during the initial stage. Lepidocrocite did not completely transform into goethite under anoxic condition with pH about 5.5 within 30 days. Fe^2+^ exhibited much higher catalytic activity than Mn^2+^ during the transformation from amorphous Fe(III)-hydroxide to lepidocrocite and goethite under anoxic conditions. The release rates of Mn^2+^ were compared to estimate the redox rates of birnessite and Fe^2+^ under different conditions.

**Conclusions:**

Redox rate was found to be controlled by chemical reaction, and increased with increasing Fe^2+^ concentration, pH, and temperature. The formation of ferric (hydr)oxides precipitate inhibited the further reduction of birnessite. The presence of air accelerated the oxidation of Fe^2+^ to ferric oxides and facilitated the chemical stability of birnessite, which was not completely reduced and dissolved after 18 days. As for the oxidation of aqueous ferrous ions by oxygen in air, low and high pHs facilitated the formation of goethite and lepidocrocite, respectively. The experimental results illustrated the single and combined effects of manganese oxide and air on the transformation of Fe^2+^ to ferric oxides.

**Graphical abstract:**
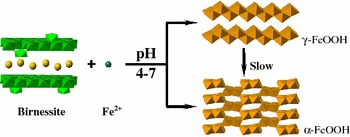
Lepidocrocite and goethite were formed during the interaction of ferrous ion and birnessite at pH 4-7. Redox rate was controlled by the adsorption of Fe2+ on the surface of birnessite. The presence of air (oxygen) accelerated the oxidation of Fe2+ to ferric oxides and facilitated the chemical stability of birnessite

## Background

The iron redox cycling, particularly Fe^II^–Fe^III^ (Fe^III^ and Fe^III^ (hydr)oxides), in soils and sediments has obtained a growing concern in the field of soil science, environmental science, and biology, as the redox of Fe^II^–Fe^III−^ occurs through either abiotic or biotic pathway in soluble, adsorbed, and solid states [1]. The redox reaction of Fe^II^–Fe^III−^ affects the geochemical behavior of minerals and nutrient elements. New experimental evidence shows that Fe^2+^ induced the release of structural manganese from manganese-doped goethite due to iron oxide recrystallization [2]. Fe(II) can also catalyze the phase transformation from ferrihydrite to goethite and hematite by electron transfer through a dissolution-reprecipitation process [3, 4]. NO_3_^−^ can be reduced by Fe(II) to form NH_4_^+^ and magnetite with nitrogen cycle in soils and groundwaters [5]. The migration and transformation of pollutants are usually coupled with the redox of Fe^II^–Fe^III−^. For example, nitrate strongly influenced arsenic cycling by oxidizing ferrous iron to produce As-adsorbing particulate hydrous ferric oxides under anoxic conditions [6]. The reduction of U(VI) and the oxidation of ferrous sulfides including pyrrhotite and pyrite are usually catalyzed by Fe^2+^ as Fe^II^–Fe^III−^ cycling works as electron transporter [7–9]. Fe-oxide formation and further transformation affect the uptake and release of cerium and uranium in Fe(II/III) aqueous solutions, and it is also found that trivalent actinides and lanthanides are released when dissimilatory iron reduction of Fe(III)-oxides leads to the formation of green rust. However, under oxidizing conditions, green rust may influence radionuclide mobility by catalyzing their transformation to a higher oxidation state [10]. These redox cycles of Fe^II^–Fe^III−^ were sometimes derived by biogeochemical pathway [1, 11]. Therefore, a comprehensive attention has been given for the oxidation of Fe(II) in environment system.

As one of the most important natural constituents in soils and sediments, manganese oxide minerals with various crystal structures exhibit excellent oxidation and adsorption capacity for organic pollutants and toxic metallic ions [6, 9, 12–16]. Manganese oxides are determined to participate in the oxidation of ferrous ions and the formation of ferric oxides [6, 13, 17]. Fe(II) usually works as catalyst and electron transfer mediator in the oxidation of ferrous sulfides and others [6, 9]. However, there are few researches on the interaction process and mechanisms of ferrous ions and manganese oxides, particularly birnessite.

Manganese oxides participated in the oxidation of ferrous ions to form ferric oxides. The oxidation rates of ferrous ions by pyrolusite and γ-MnO_2_ were compared in sulfate solution, and the influence factors of acidity and particle size were considered, indicating that oxidation rate increased about 2 times when pyrolusite was substituted by γ-MnO_2_ [18]. Birnessite, formed from microbial oxidation of Mn(II), is often enriched with heavy metals and alkaline and alkali earth metals, and generally exhibits the highest oxidation activity and largest adsorption capacity [2, 12, 13, 16, 19]. The oxidation of Fe(II), such as ferrocyanide and Fe^2+^, by birnessite was also studied, and the influence of pH and temperature on the release rate of Mn^2+^ was considered [20, 21]. Column experiment was conducted to study the reduction of Mn-oxides by ferrous ions in a flow system, and reactive transport modeling was used to analyze, quantify, and elucidate the different reaction controls and their interaction, and it was found that the release of newly formed Fe^3+^ and Mn^2+^ would be retarded as they would be first adsorbed on the surface of manganese oxides [22]. During the redox process, initial reduction rates were much faster than long-term rates because of the inhibition by Fe(III) precipitates in the later stage at higher pH [23]. The above researches focus on reaction kinetics, which is usually based on the deduced reaction process. However, limited information is available for the interaction process and mechanisms of ferrous ions and manganese oxides, especially for the transformation processes of ferric oxides.

Transition metal ions affected the transformation of ferric oxides with different crystal structures. The presence of Ni(II) and Pb(II) inhibited the transformation of amorphous iron oxide into a more crystalline form [24, 25]. Mn(II) participates in the transformation of ferric oxides, because Mn(II) substitution would increase the cell volumes and decrease the degree of crystallinity of goethite; in addition, dissolution and recrystallization subsequently occurred [26]. During the oxidation of ferrous ions by birnessite, Mn(II) would be released and affect the formation process of ferric oxides. The further transformation of ferric oxides and manganese oxides needs to be intensively studied in detail, especially with regard to the influence of the released Mn(II) and Fe(II) on the formation of ferric oxides.

In the supergene environment, oxygen usually participates in the redox behaviors of active substances. The adsorbed Mn(II) could also catalyze the oxidation of Cr(OH)_3_(s) to toxic Cr(VI) in air [27]. The presence of oxygen improves the chemical stability of manganese oxides during the reduction process [28]. Sometimes, this redox reaction was only tested in acidic environment with anoxic conditions. The influence of air (oxygen) on reaction process and kinetics of birnessite and ferrous ions was seldom investigated. Because the redox reactions usually occur in open systems, the participation of air also needs to be further studied.

This work aims for the better understanding of the interaction mechanisms and kinetics of ferrous ion and hexagonal birnessite in aqueous systems. The transformation of synthesized birnessite and ferrous ions during the reaction process and the influences pH, temperature, and concentration on reaction rate were studied. The admission of air into reaction solution is to simulate the aerobic environment in open system. The catalysis of Fe^2+^ and Mn^2+^ in the transformation from amorphous Fe(III)-hydroxide to lepidocrocite and goethite were also investigated in nitrogen atmosphere.

## Experimental

### The synthesis of birnessite

Birnessite was synthesized through the reduction of potassium permanganate by concentrated hydrochloric acid [29]. KMnO_4_ of 31.61 g was dissolved in 300 mL deionized water in a conical flask and boiled with an oil-bath heated at 100 °C, and then 60 mL of 6 mol L^−1^ hydrochloric acid was added dropwisely to the boiling solution at 0.7 mL min^−1^ with vigorous stirring. The reaction lasted for 30 min, and then the suspension was aged for 12 h at 60 °C. The as-obtained mineral was washed with deionized water until filtrate conductivity was below 20.0 μS cm^−1^, and subsequently dried in an oven at 60 °C, and birnessite was prepared and used in the subsequent redox experiments.

### The redox of birnessite and ferrous ions

FeSO_4_·7H_2_O of 0.5561, 1.1121 and 2.2242 g was dissolved into 200 mL distilled deionized water, and the concentration of Fe^2+^ was about 10, 20, and 40 mmol L^−1^ (mM), respectively. The reactor was continuously purged with high purity nitrogen gas (99.999 %, Wuhan Iron and Steel (Group) Corp., China). Nitrogen pressure inside the reactor was maintained to be slightly higher than atmospheric pressure to further prevent air ingress. The as-prepared birnessite of 0.2 g was then added to FeSO_4_ solution with stirring. The pHs of reaction systems were controlled at 4.0, 5.5 and 7.0, respectively, using NaOH and H_2_SO_4_ of 1.0 mol L^−1^. After a period of reaction, about 3.0 mL solution was filtered through a 0.22-μm microporous membrane filter. The solid products and dissolved components in filtrate were respectively characterized to analyze reaction mechanism. To simulate the open system, air was admitted into the reaction solution instead of nitrogen.

To study the influence of Fe^2+^ and Mn^2+^ on the formation and further transformation of ferric oxides, Fe_2_(SO_4_)_3_ of 8.0 g was dissolved into 200 mL distilled deionized water in nitrogen atmosphere, and MnSO_4_ or FeSO_4_ was then added at a concentration of 10 mmol L^−1^, respectively, and pH was controlled at 5.5. To investigate the oxidation process of ferrous ions in the presence of air, air was admitted into 200 mL FeSO_4_ solution of 20 mmol L^−1^ at pH 5.5 and pH 7.0, respectively. After filtration, the filtrates and precipitates were respectively characterized at different times.

### Characterization methods

The concentration of ferrous ions in filtrate was directly determined by ultraviolet–visible spectrophotometry (UV-1800, Shanghai Mapada Instruments Co., Ltd., China). The filtrate could be first reduced by oxammonium hydrochloride, and then Fe^2+^ concentration was measured as the total concentration of Fe^2+^ and Fe^3+^, and Fe^3+^ concentration could be obtained by subtraction. The total concentration of Fe^2+^ and Fe^3+^ in reaction system and manganese content in samples were also quantified by atomic absorption spectroscopy (Varian AAS240FS). The average oxidation state (AOS) of manganese was measured by an oxalic method [30]. The content of K in samples and released K^+^ concentration in filtrate was determined by flame photometer (HG-3 blaze photometer). BET surface area of as-obtained birnessite was analyzed by Micromeritics ASAP2020 using nitrogen adsorption measurements. All chemical analysis for each sample was repeated three times.

After a period of reaction time, about 3.0 mL solution in reaction system was drawn off and filtered through a 0.45-μm microporous membrane filter. The wet solid products were soon identified by X-ray diffraction spectrometry (XRD, Bruker D8 Advance diffractometer with Cu Kα) at a scan rate of 4° min^−1^. Before and after redox reactions, their morphologies were characterized by scanning electron microscopy (SEM, JEOL, JSM-6700F Field Emission) and transmission electron microscopy (TEM, Hitachi, H-7650). Fourier transform infrared spectroscopy (FTIR, Nicolet 8700) was used to characterize the functional group of redox products with a DTGS detector by making pellets with KBr powder. The contents of crystal water in birnessite were calculated from the mass balance using thermo-gravimetric analysis (TGA) with NETZSCH TG 209 thermal analysis system.

## Results and discussion

### The oxidation of Fe^2+^ by birnessite under anoxic condition

**Fig. 1:**
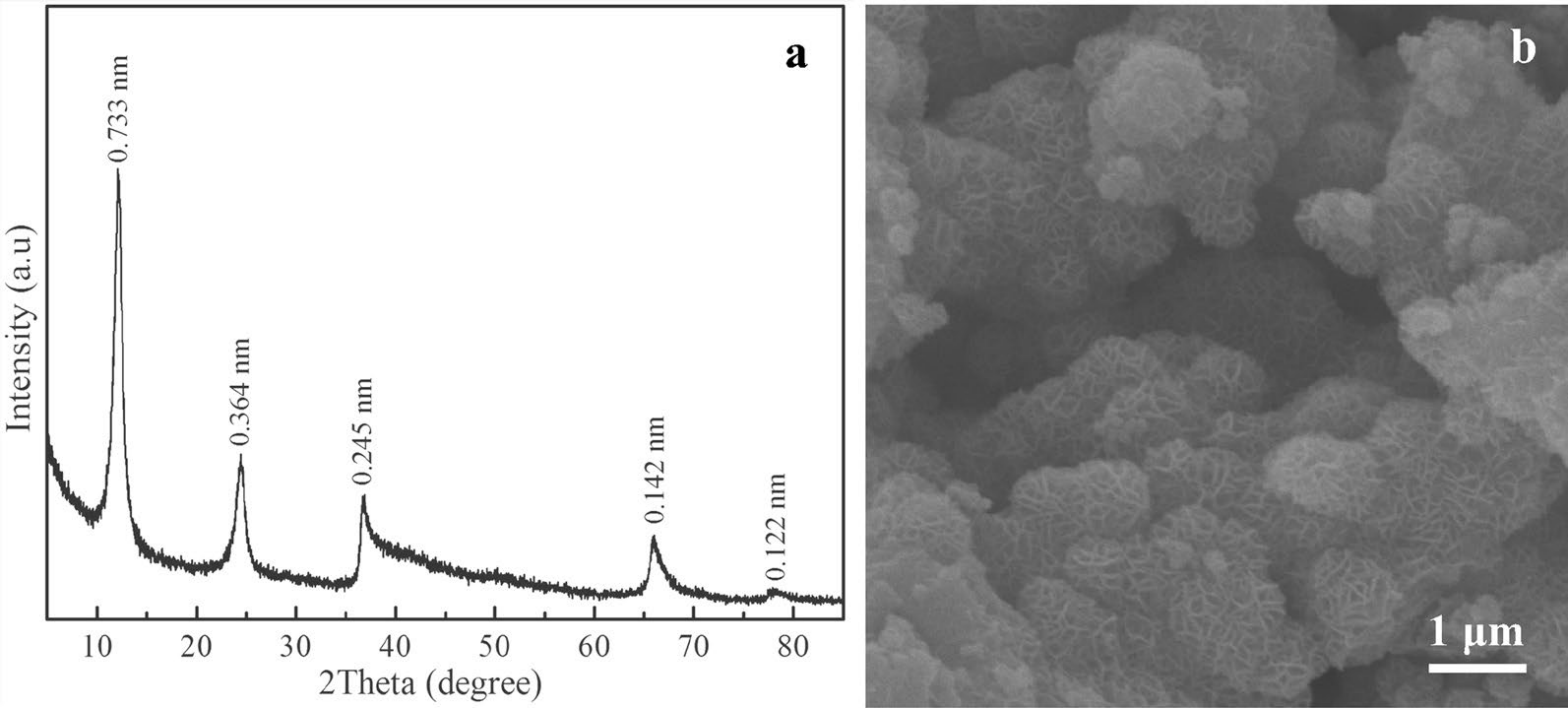
XRD patterns (**a**) and SEM image (**b**) of synthesized birnessite dried at 60 °C for 24 h

Figure 1a shows the XRD patterns of as-prepared hexagonal birnessite, and the detectable five peaks were consistent with JCPDS No. 86-0666, indicating that pure-phased birnessite was formed. As for hexagonal birnessite, typical three-dimension hierarchical microspheres composed of disk-shaped plates were observed as shown in Fig. 1b. The AOS of birnessite was titrated to be 3.85, and the chemical formula for birnessite was defined as K_0.23_MnO_2.03_·0.6H_2_O. The specific surface area of as-obtained birnessite was found to be 27.8 m^2^ g^−1^.

**Fig. 2:**
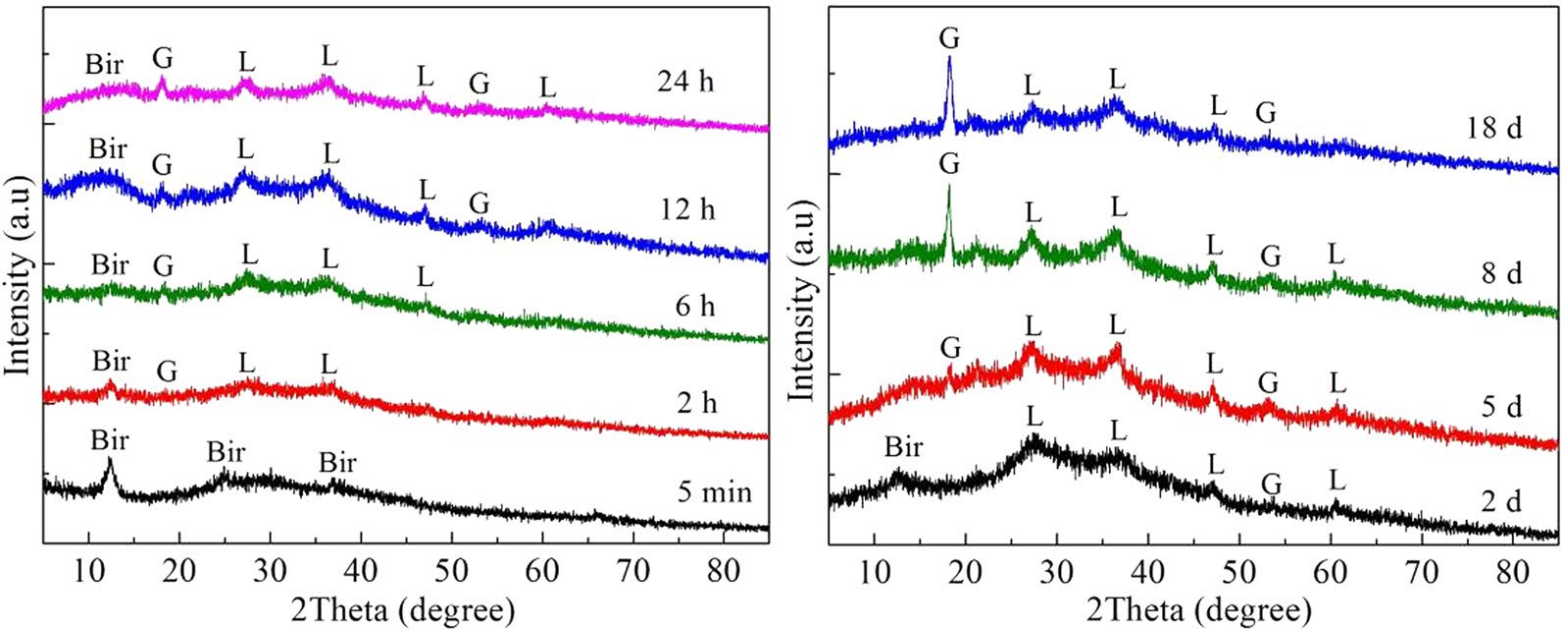
XRD patterns of solid products of 20 mM Fe^2+^ oxidized by 1.0 g L^−1^ birnessite with pH 5.5 in nitrogen atmosphere at different times

The redox reaction was conducted in nitrogen atmosphere to create an anoxic condition at pH 5.5, and solid products were characterized by XRD at different times as shown in Fig. 2. After 5 min of reaction, a mixture of birnessite and amorphous ferric oxides was formed, and then lepidocrocite (γ-FeOOH, JCPDS No. 76-2301) was formed after 2 h. Birnessite was completely reduced due to the disappearance of characteristic diffraction peaks after 2 days as shown in Fig. 2. The increase of diffraction peaks of goethite (α-FeOOH, JCPDS No. 81-0464) and a relative weakening of lepidocrocite is possibly due to the transformation from lepidocrocite to goethite after 8 days. The transformation from lepidocrocite to goethite in the nucleation process was influenced by multiple factors [31, 32]. This transformation would be inhibited by traces of silicate, aluminate and stannate [32], and markedly interfered by Ti(IV), Cu(II), Cr(III) and Ni(II), and the coexistence of Fe(II) and SO_4_^2-^ is necessary for this transformation [31]. In the current system, both SO_4_^2−^ and Fe^2+^ participated in the reaction, and the transformation from lepidocrocite to goethite occurred. However, the transformation from relatively metastable lepidocrocite to goethite is extremely slow at ambient temperature [33]. The presence of Mn^2+^ and other transition metal ions likely assisted this progress, and the newly released Mn^2+^ could incorporate in goethite [2, 31–33]. Conversely, aqueous Fe^2+^ can induce the release of structural manganese from manganese-doped goethite [2].

**Fig. 3:**
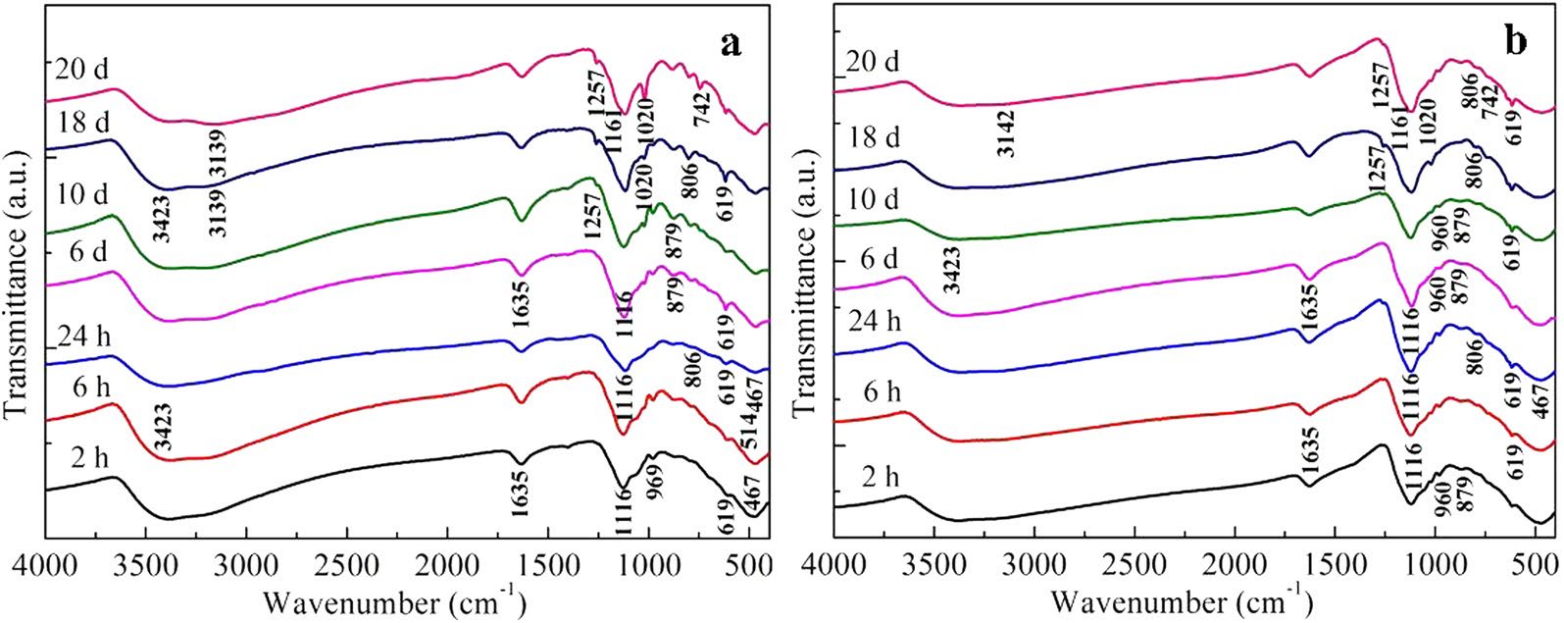
FTIR spectra of solid products of 20 mM Fe^2+^ oxidized by 1.0 g L^−1^ birnessite with pH 5.5 in nitrogen atmosphere (**a**) and air (**b**) at different times

The chemical compositions of solid products were further characterized using FTIR spectroscopy as shown in Fig. 3a. These bands at 467 and 514 cm^−1^ were assigned to Mn–O lattice vibrations of birnessite, and the absorption band around 969 cm^−1^ was likely due to the bending vibration of Mn(III/IV)OH, which faded and disappeared after 10 days owing to the complete reduction and dissolve of birnessite [34, 35]. The dominant absorption peaks at 1635 and 3423 cm^−1^ were assigned to the stretching and bending vibrations of crystal water and adsorbed water, respectively [35]. These absorption peaks at 806, 1020, 1116 cm^−1^ are attributed to lepidocrocite, and peaks at 619, 879, and 1020 cm^−1^ are assigned to goethite [31, 36]. A dominant absorption band at 3139 cm^−1^ was possibly due to the vibration of hydroxyl group in α-FeOOH [36]. The increase in the intensity of peaks at 619, 879, 1020, and 3139 cm^−1^ suggested an increase in the content of goethite in solid products. The results further suggested that an incomplete transformation occurred and a mixed phase of lepidocrocite and goethite was formed.

**Fig. 4:**
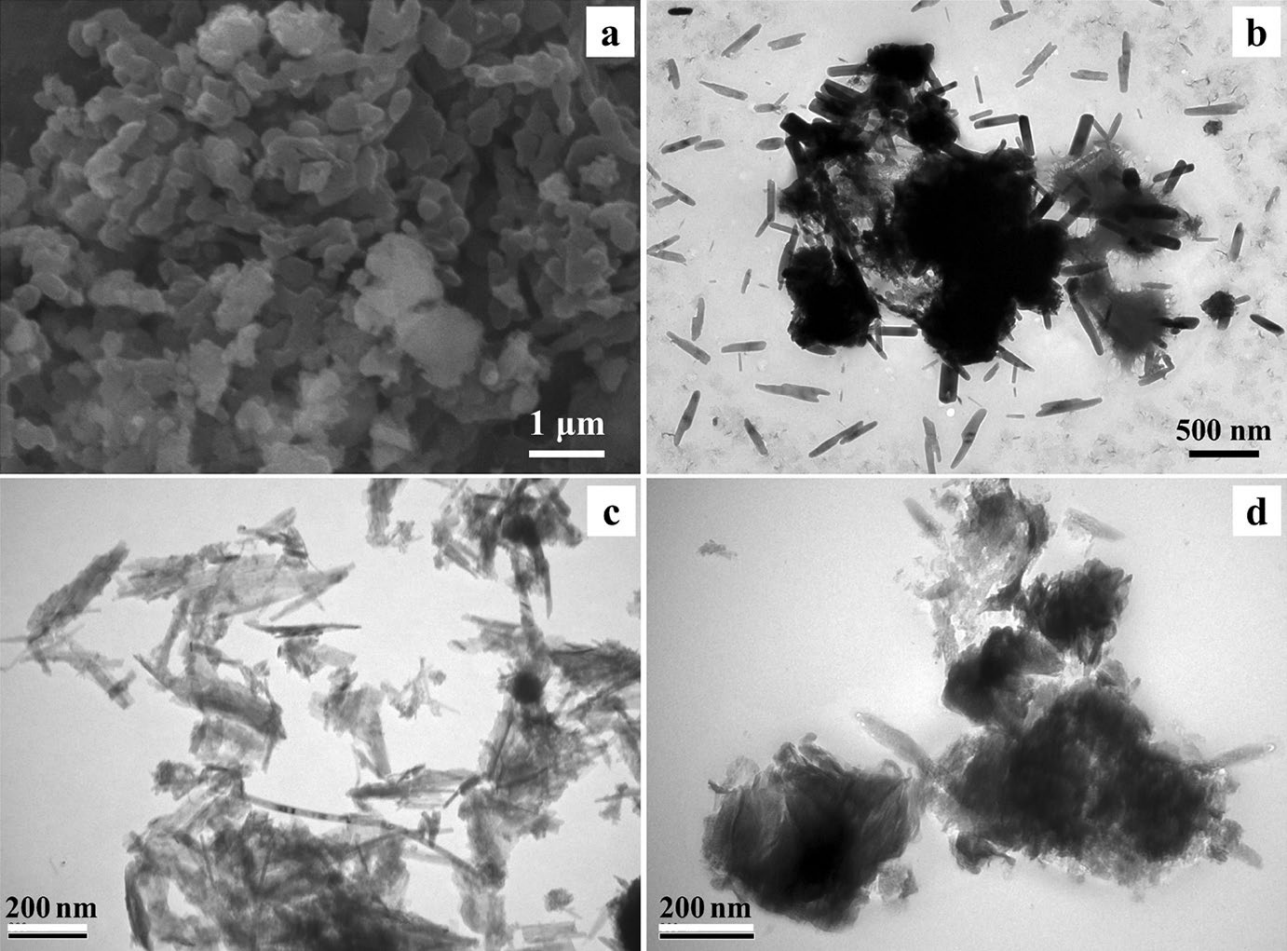
SEM and TEM images of solid products of 20 mM Fe^2+^ oxidized by 1.0 g L^−1^ birnessite at 12 h (**a**, **b**) and 20 days (**c**) in nitrogen atmosphere, and in air at 20 days (**d**)

The transformation process was further verified by the change of particle morphology observed by SEM and TEM as shown in Fig. 4a–c. Flower-like birnessite and uniform platelet lepidocrocite particles were observed after 12 h of reaction (Fig. 4a), which was further confirmed by TEM after ultrasonic dispersion, and goethite was almost absent (Fig. 4b). These results implied that lepidocrocite was formed during the initial stage. After 20 days, the proportion of needle-like goethite particles increased, and flower-like birnessite particles disappeared (Fig. 4c). The similar morphologies were observed during the transformation of lepidocrocite to goethite [31, 32, 37]. These results further indicated that this transformation was too slow to obtain single-phased goethite.

**Fig. 5:**
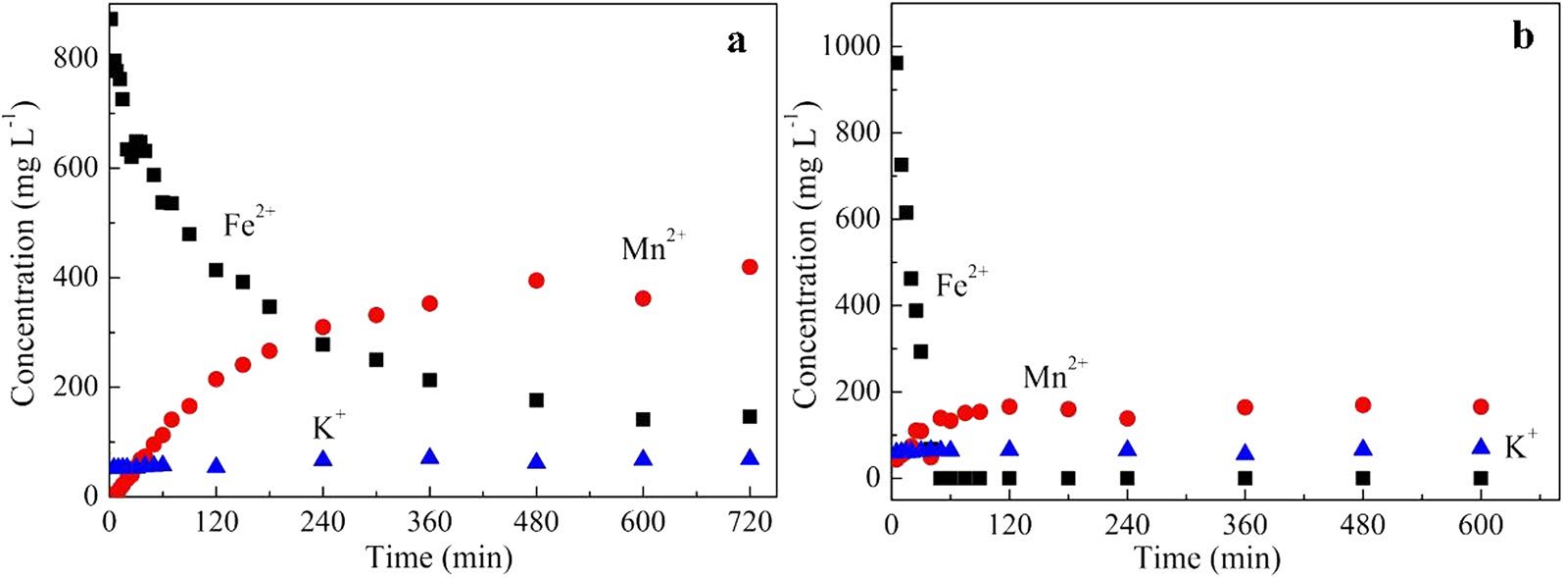
The concentration of Fe^2+^, Mn^2+^ and K^+^ in reaction system of 20 mM Fe^2+^ oxidized by 1.0 g L^−1^ birnessite with pH 5.5 in nitrogen atmosphere (**a**) and in air (**b**) at different times

During the reaction process, the concentration of Fe^2+^, Mn^2+^ and K^+^ was quantified as shown in Fig. 5a. It was observed that Fe^2+^ concentration decreased significantly from 1120 mg L^−1^ in the initial stage to about 150 mg L^−1^ at 720 min, and the released Mn^2+^ and K^+^ concentration increased to 420 and 68.5 mg L^−1^, respectively, at 720 min. Organic matter, silicate, phosphate, and metal ions also participate in the formation of ferric oxides and exhibit different effects on transformation rate [31, 32, 37]. The presence of Fe^2+^ and Mn^2+^ might participate in the transformation of ferric oxides including lepidocrocite and goethite. Fe^2+^ works as electron mediators and accelerate the formation of goethite, however, the other metal ions, such as Ti^4+^, Cu^2+^ and Cr^3+^, effectively interfere with the transformation for the interruption of electron transfer [10, 31]. In order to study the influence of Fe^2+^ and Mn^2+^ on the transformation of ferric oxides, Fe^2+^ and Mn^2+^ were added to Fe_2_(SO_4_)_3_ solution with pH 5.5 and solid products were analyzed.

**Fig. 6:**
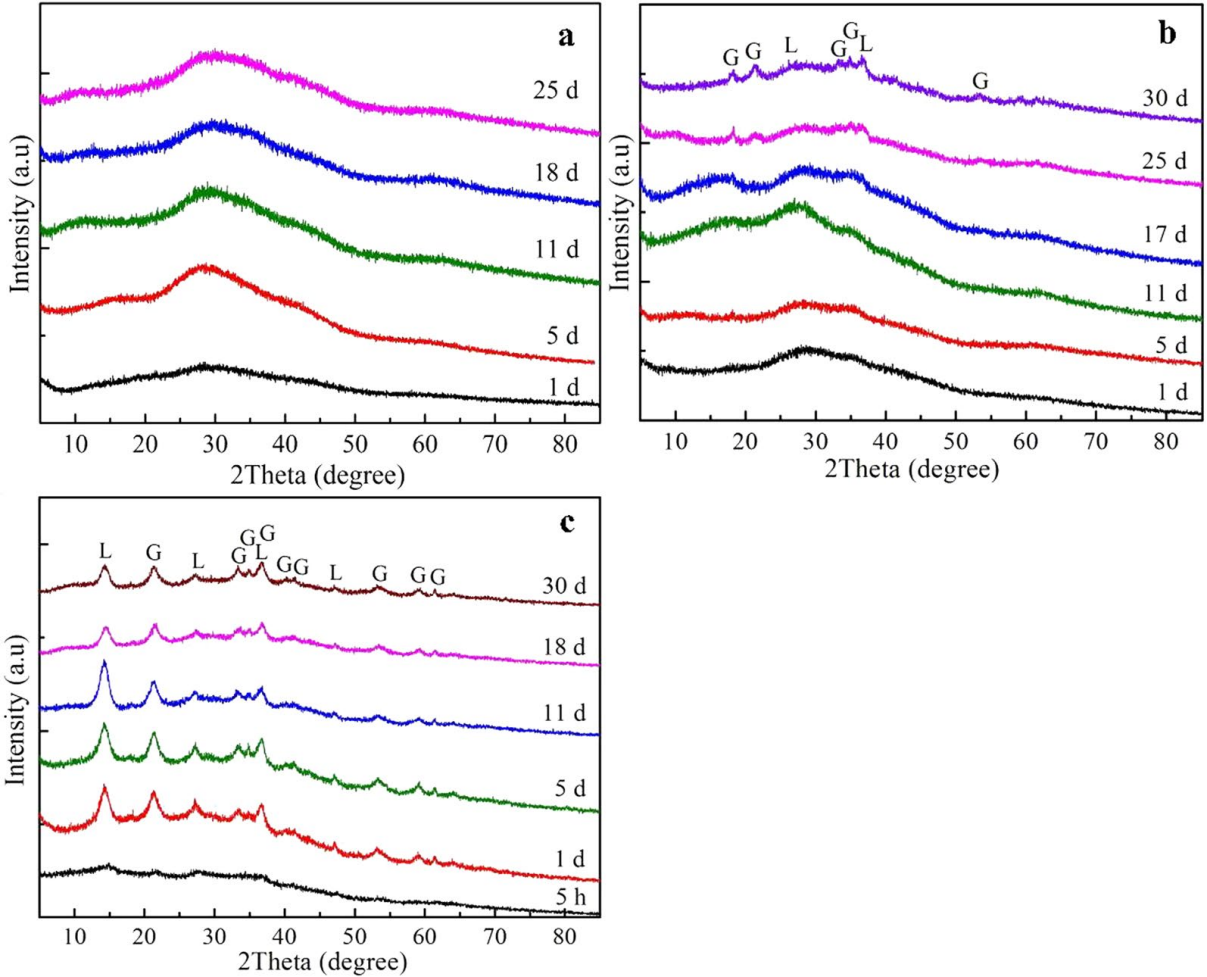
XRD patterns of solid products of 10 mM Fe_2_(SO_4_)_3_ (**a**), Fe_2_(SO_4_)_3_ (10 mM)/MnSO_4_ (10 mM) (**b**), and Fe_2_(SO_4_)_3_ (10 mM)/FeSO_4_ (10 mM) (**c**) aqueous solutions in nitrogen atmosphere with pH 5.5 at different times

Figure 6 shows the XRD patterns of solid products of Fe_2_(SO_4_)_3_ (10 mmol L^−1^), Fe_2_(SO_4_)_3_ (10 mmol L^−1^)/MnSO_4_ (10 mmol L^−1^), and Fe_2_(SO_4_)_3_ (10 mmol L^−1^)/FeSO_4_ (10 mmol L^−1^) aqueous solutions in nitrogen atmosphere with pH 5.5 at different times. As for Fe_2_(SO_4_)_3_ aqueous solution, amorphous ferric (hydr)oxide was formed at pH 5.5, and its crystallinity did not obviously increase after 25 days. The addition of Mn^2+^ facilitated the formation of a mixture of lepidocrocite and goethite, and the latter was major product after 25 days (Fig. 6b). The presence of Fe^2+^ significantly accelerated the formation of lepidocrocite and goethite, which were observed after 1 days (Fig. 6c). Lepidocrocite was the major product in the initial stage, and would slowly transform into goethite, which was indicated by the relative change in the intensity of XRD peaks. These results exhibited that Fe^2+^ had higher catalytic activity than Mn^2+^ for the formation of crystalline ferric oxides, and further suggested that not all transition metal ions inhibited the formation of goethite from lepidocrocite, which was likely due to the particular affinity and constant pH during this reaction process [2, 31, 38].

### The oxidation of Fe^2+^ by birnessite in air

**Fig. 7:**
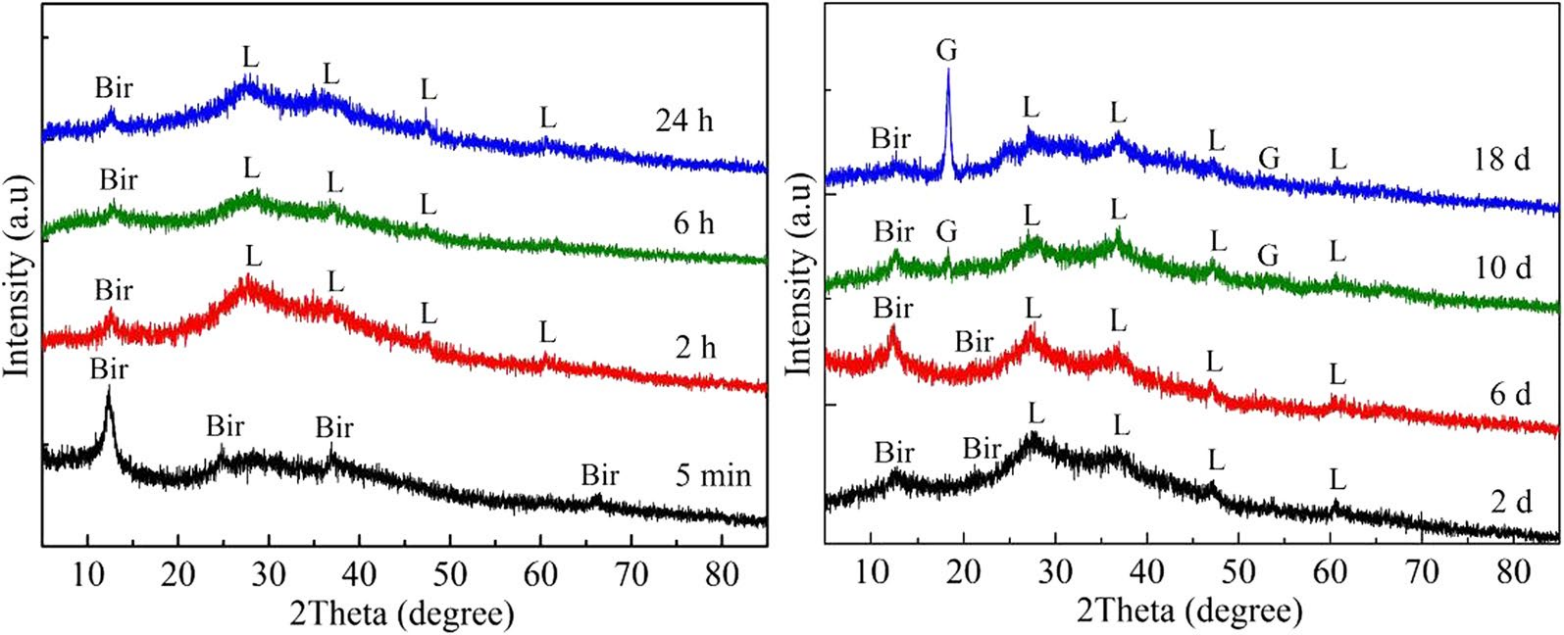
XRD patterns of solid products of 20 mM Fe^2+^ oxidized by 1.0 g L^−1^ birnessite in air at different times

In soils and sediments, redox reactions are usually driven by oxygen, ferric irons, manganese oxides and microorganisms [8, 39, 40]. To simulate the abiotic oxidation behavior of Fe^2+^ by birnessite in an open system, air was admitted into the reaction system, and intermediate products were characterized. As shown in Fig. 7, a mixture of birnessite and lepidocrocite was produced within 6 days, and then goethite was formed after 10 days. The fact that the participation of oxygen improved the chemical stability of birnessite was further verified by the concentration of released Mn^2+^ as shown in Fig. 5b. The remove rate of Fe^2+^ concentration significantly increased, compared with the reaction under anoxic condition, and Fe^2+^ concentration decreased to about zero at 1 h (Fig. 5). However, the concentration of generated Mn^2+^ and K^+^ just reached about 170 and 70 mg L^−1^ after 6 h, respectively. These results indicated the oxidation of Fe^2+^ by oxygen and the improvement of the chemical stability of birnessite. The chemical stability of birnessite was improved in the presence of oxygen, likely due to the fact that the newly formed Mn(III) from Mn(IV) in birnessite would be re-oxidized by oxygen in air [28, 30, 34, 39, 41]. In our previous work, during the oxidation process of soluble sulfide by todorokite and oxygen, the reaction rate was controlled by the rate of diffusion of soluble sulfide and todorokite, and the admission of oxygen reduced the initial oxidation rate of soluble sulfide by todorokite due to the decrease of active Mn(III) content in manganese oxide owing to the oxidation of Mn(III) to Mn(IV) by oxygen [28]. On the other hand, oxygen would directly oxidize ferrous ions, and the consumption of oxidant birnessite decreased in the same reaction system. The improved redox stability of birnessite was further confirmed by TEM image as shown in Fig. 4d. Although the morphologies of birnessite became pulverized and indistinct, the aggregates could be observed. These particles were completely dissolved and unobservable as the redox reaction occurred in nitrogen atmosphere for the same time (Fig. 4c). There was no obvious change for the concentration of released K^+^ likely due to ion-exchange by Fe^3+/2+^ and H^+^ during the initial stage and the complete reduction and dissolve during the final stages.

**Fig. 8:**
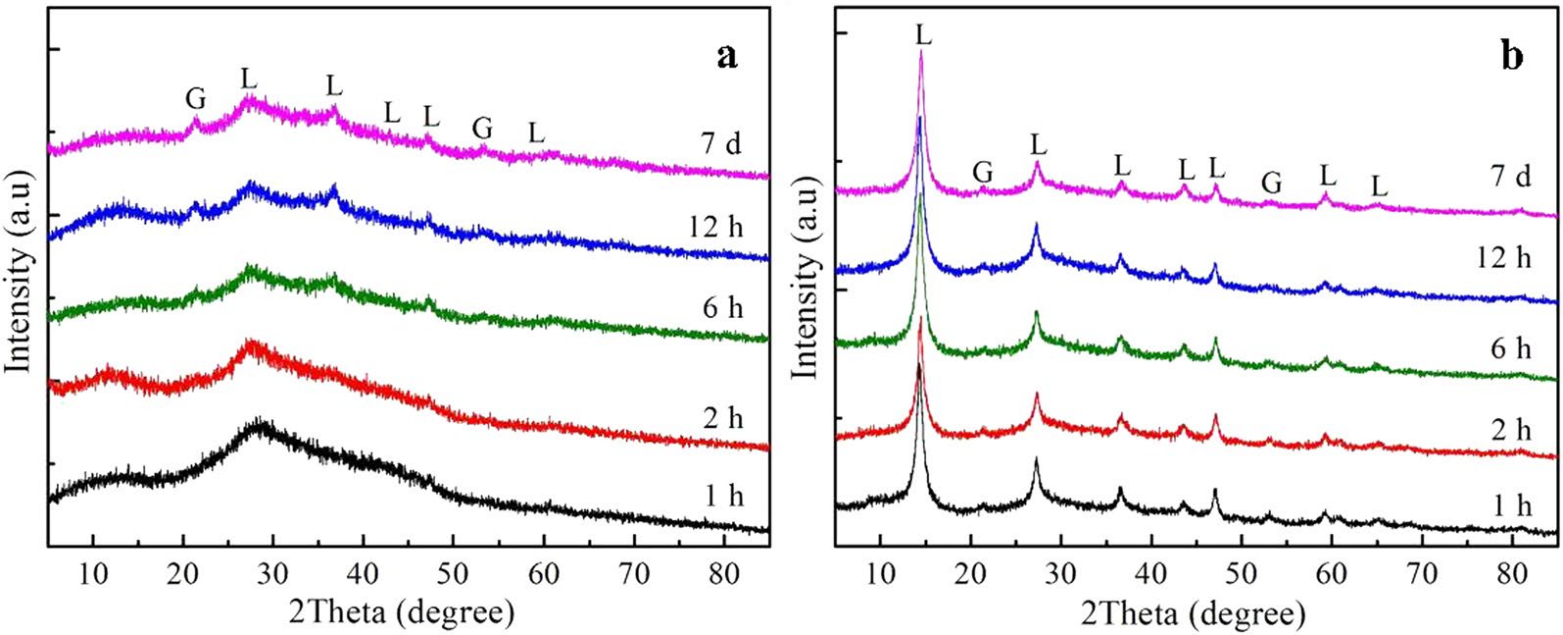
XRD patterns of solid products of 20 mM FeSO_4_ oxidized by air with pH 5.5 (**a**) and pH 7.0 (**b**) at different times

In order to further investigate the influence of oxygen on the transformation process of ferrous ions to ferric oxides, air was directly pumped into aqueous solution containing FeSO_4_ of 20 mmol L^−1^ at pH of 5.5 and 7.0, respectively. Figure 8 shows the XRD patterns of solid products at different times. Amorphous ferric oxide was first formed and then transformed into a mixture of lepidocrocite and goethite with low degree of crystallinity when reaction system was controlled at pH 5.5. When pH was adjusted to 7.0, crystalline lepidocrocite was formed with a small amount of weak crystalline goethite after 1 h. The relative content of lepidocrocite and goethite had no significant changes after 7 days. In this work, pH played an important role in the transformation of ferric oxides. The formation of FeOH^+^ accelerated the dissolution of amorphous ferric oxide at pH 7.0, which facilitated the transformation of lepidocrocite during the dissolution/reprecipitation process induced by Fe^2+^ [3, 38]. As pH was less than 5.0, single-phased goethite could be formed in this system (figure not shown), which further indicated the transformation process from lepidocrocite to goethite.

**Fig. 9:**
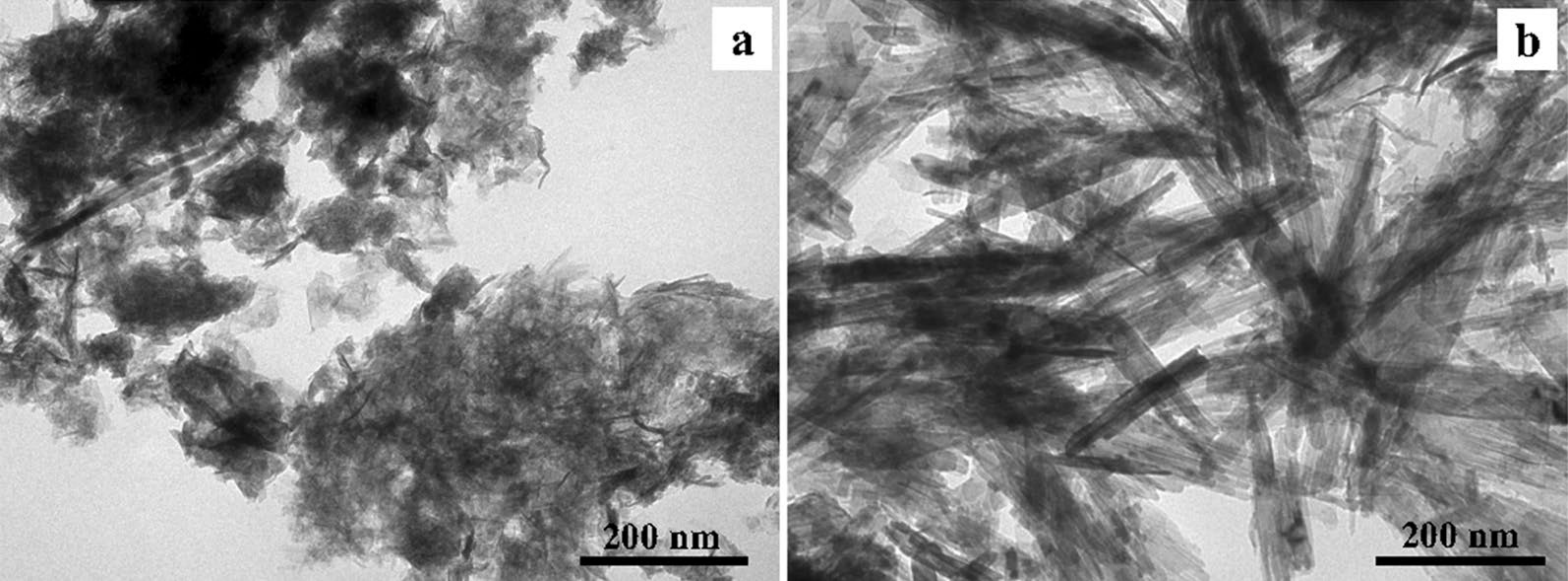
TEM images of solid product of 20 mM FeSO_4_ oxidized by air at pH 5.5 (**a**) and pH 7.0 (**b**) for 7 days

The morphologies of the solid products were characterized by TEM as shown in Fig. 9. Needle-like goethite and amorphous particles within 100 nm were formed when aqueous FeSO_4_ oxidized by air at pH 5.5 for 7 d. As pH increased to 7.0, homogeneous platelet particles of lepidocrocite were formed with particle size more than 200 nm. These results are consistent with the XRD identification (Fig. 8), further indicated the influence of oxygen in air on the oxidation rate of Fe^2+^ and the reciprocal transformation of ferric oxides, such as goethite and lepidocrocite.

### The oxidation kinetics of Fe^2+^ by birnessite

**Fig. 10:**
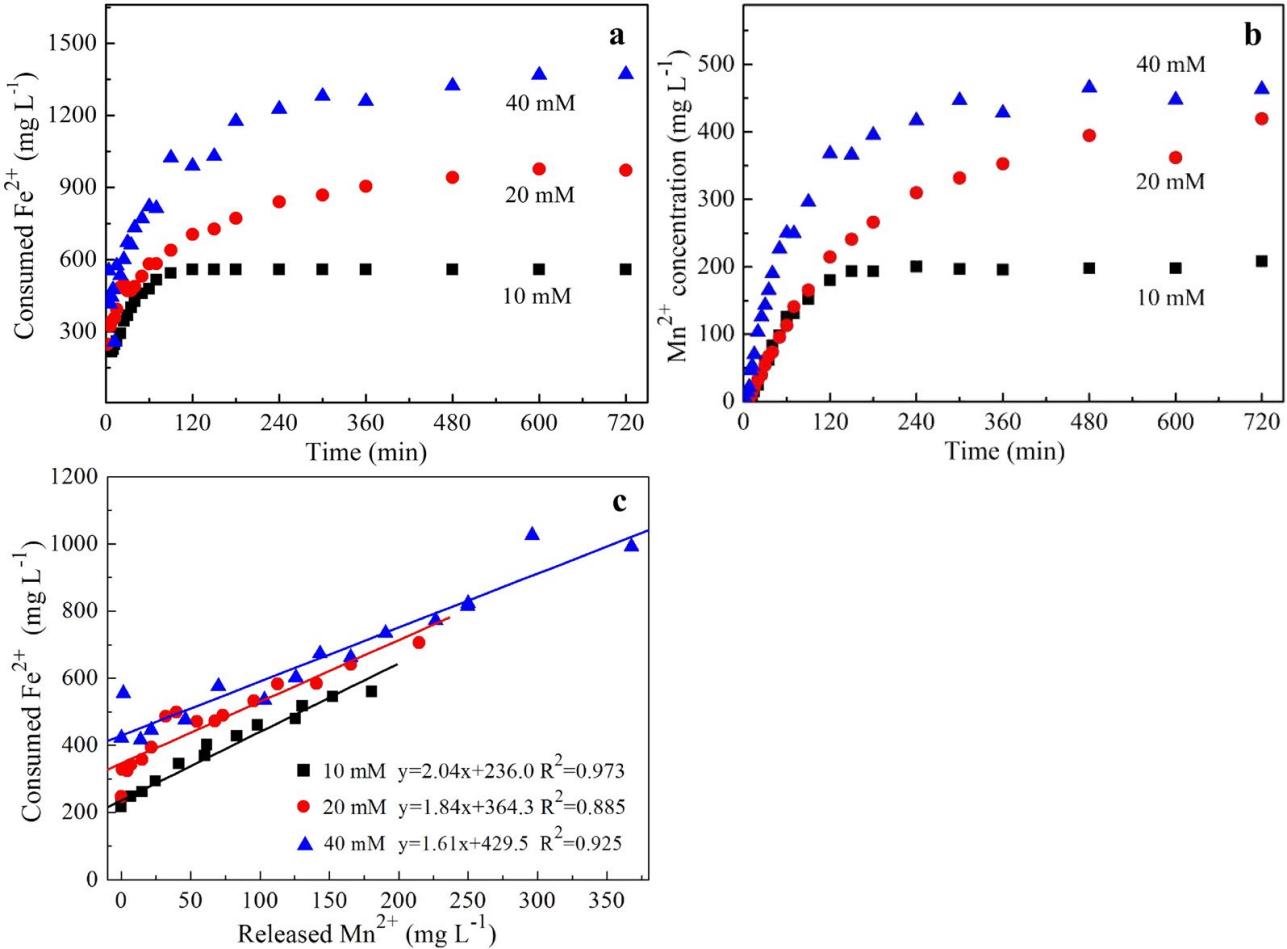
Concentration of consumed Fe^2+^ with different initial concentration (**a**) and released Mn^2+^ concentration (**b**) and the relationship of consumed Fe^2+^ and released Mn^2+^ concentration in reaction system within 120 min (**c**)

The influential factors on the reaction rate of ferrous ions oxidation by birnessite were considered including Fe^2+^ concentration, pH, temperature, and oxygen in air. As shown in Fig. 10a, when Fe^2+^ ion concentration was controlled at 10, 20, and 40 mmol L^−1^, after 12 h of reaction, the concentration of consumed Fe^2+^ reached about 559, 705, and 990 mg L^−1^, which corresponding consumption rates approaching to 100, 87 and 61 %, respectively. The amount of released Mn^2+^ can be used to indicate the redox rate [18, 20, 21, 42]. In this work, higher concentration of reactant facilitated the larger capacity for Fe^2+^ oxidation. Mn(III) complexes would not be formed, and dissolved Mn^2+^ ions and ferric (hydr)oxides including goethite and lepidocrocite were major products [20, 42]. The redox reaction may be represented as follows:

1 $${\text{K}}_{0. 2 3} {\text{MnO}}_{ 2.0 3} { \cdot }0. 6 {\text{H}}_{ 2} {\text{O}} + 1. 8 3 {\text{ Fe}}^{ 2+ } + 1.0 3 {\text{ H}}_{ 2} {\text{O}} \to {\text{Mn}}^{ 2+ } + 1. 8 3 {\text{ FeOOH}} + 0. 2 3\,\, {\text{K}}^{ + } + 1. 4 3 {\text{ H}}^{ + }$$

As reported, the generation of FeOOH could not be confirmed by XRD [20], however, the transformation process of ferric (hydr)oxides was characterized and illustrated in this work. According to reaction (1), the molar ratio of the concentration of oxidized Fe^2+^ to that of the released Mn^2+^ would be 1.83 (mass ratio: 1.86). In this experiment, the removal Fe^2+^ in reaction solution was all treated as the oxidized Fe(III), which existed as adsorption states on the surface of manganese oxides and the precipitate of ferric (hydr)oxides including goethite and lepidocrocite. The interrelation of the concentration of oxidized Fe^2+^ and the released Mn^2+^ was compared and the influence of reaction conditions was studied.

Figure 10b shows the concentration of released Mn^2+^ as the reaction proceeded. In the initial stage, the release rate of Mn^2+^ was similar when Fe^2+^ of 10 and 20 mmol L^−1^ was oxidized by birnessite of 1.0 g L^−1^, and it significantly increased when Fe^2+^ of 40 mmol L^−1^ was used instead. The concentration of released Mn^2+^ could reach 208, 420, and 463 mg L^−1^, respectively, after reaction of 12 h. The amount of the released Mn^2+^ increased with an increase in the concentration of ferrous ions, suggesting that high concentration of Fe^2+^ accelerated the reduction of birnessite. The mass ratios of Fe^2+^ consumption to Mn^2+^ production (ΔFe/ΔMn slopes) were 2.04, 1.84, and 1.61 when ferrous ion concentration was controlled at 10, 20, and 40 mmol L^−1^, respectively (Fig. 10c).

During the reduction process of birnessite, active adsorption sites would first be quickly occupied by Fe^2+^, and then redox reactions occurred. Subsequently, the newly formed Mn^2+^ from the reduction of birnessite would be paritially released into aqueous system, because some Mn^2+^ ions would be readsorbed on the surface of newly exposed birnessite if incomplete redox occurred. We have compared the theoretical and experimental concentrations of consumed Fe^2+^ and released Mn^2+^ for the reactions of Fe^2+^ of 10, 20 and 40 mmol L^−1^ and birnessite of 1 g L^−1^, respectively. When Fe^2+^ of 10 mmol L^−1^ participated in the reaction, 5.46 mmol L^−1^ Mn^2+^ should be released for the complete oxidation of Fe^2+^ by excessive birnessite. When Fe^2+^ concentrations were 20 and 40 mmol L^−1^, excessive Fe^2+^ was used and about 9.32 mmol L^−1^ Mn^2+^ should be released. However, the concentration of released Mn^2+^ was determined to be 3.58, 6.03 and 8.12 mmol L^−1^ after 30 min for the reactions of birnessite of 1 g L^−1^ and Fe^2+^ of 10, 20 and 40 mmol L^−1^, respectively. All the released Mn^2+^ concentrations were lower than the theoretical values. Therefore, all the reductions of birnessite by Fe^2+^ were incomplete when Fe^2+^ ions were controlled at 10, 20 and 40 mmol L^−1^, and the formation of the precipitate of ferric (hydr)oxides including goethite and lepidocrocite possibly inhibited the further reduction of birnessite [20, 22, 23, 43]. Another possibility should be considered for the low concentration of released Mn^2+^, the formed Mn^2+^ would be partially readsorbed on the surface of newly exposed birnessites, resulting in a decrease in released Mn^2+^ concentration. The similar concentrations of released Mn^2+^ were determined as Fe^2+^ concentration was controlled at 10 and 20 mmol L^−1^, suggesting that Mn^2+^ could be readsorbed because new active adsorption sites would be exposed with the partial reduction and dissolution of birnessite.

As shown in Fig. 5, released K^+^ was determined to be 1.34 mmol L^−1^ within 5 min, and K^+^ concentrations increased to 1.46 and 1.79 mmol L^−1^ after reaction of 60 and 360 min, respectively. However, complete reduction of birnessite would result in the concentration of released K^+^ about 2.15 mmol L^−1^, which further suggested that rapid adsorption in the initial stage and the incomplete reduction of birnessite occurred in this process.

Figure 10a shows the concentration of Fe^2+^ at different times as the initial concentrations of Fe^2+^ were changed from 10 to 40 mmol L^−1^ in reaction systems. The consumed Fe^2+^ concentration reached 4.0, 4.4 and 7.5 mmol L^−1^ within 2 min, and they approached to 6.6, 8.4 and 12.0 mmol L^−1^ after 30 min, respectively. However, the concentrations of released Mn^2+^ were almost zero within 2 min, and they approached to 1.1, 1.0 and 2.6 mmol L^−1^ when Fe^2+^ of 10, 20, and 40 mmol L^−1^ participated in the reaction, respectively. These results further indicated that the adsorption of Fe^2+^ on birnessite surface might be the major reaction, and high concentration of Fe^2+^ facilitated the reduction of birnessite and the corresponding release rate of Mn^2+^, which proved the redox kinetics of birnessite and ferrous ions [20, 22].

As reported, for the oxidation of Fe^2+^ and Cr^3+^ by manganese oxides, reaction rate was controlled by chemical reaction and not dependent upon diffusion from the bulk solution or transport of dissolved species from birnessite at pH > 4, and initial reduction rates can be significantly faster than long-term rates because of the inhibition by Fe(III) precipitates [20, 22, 23, 43]. In this work, the adsorption of Fe^2+^ on the surface of birnessite was extremely fast, and it could be proved by the release rate and concentration variation of K^+^. In the initial stage, almost the similar amount of Fe^2+^ would be quickly adsorbed on the surface of birnessite although the different concentrations of Fe^2+^ were applied because of the fixed concentration of oxidant birnessite. Therefore, the adsorption of Fe^2+^ played an important role in the decrease of Fe^2+^ in the initial stage, and they have the similar concentration changing trend due to the same amount of birnessite. The decrease in Fe^2+^ concentration was mainly attributed to the adsorption reaction in the initial stage. The incomplete redox reactions occurred, and redox reaction worked as the rate-determining step. The redox rate and the corresponding release rate of Mn^2+^ increased with an increase in the concentration of Fe^2+^ [20]. The change amplitude of the concentration of Fe^2+^ was not as significant as that of Mn^2+^. Therefore, the decrease of ΔFe/ΔMn values with an increase in Fe^2+^ concentration was owing to the increase redox rate as Fe^2+^ concentration increased.

**Fig. 11:**
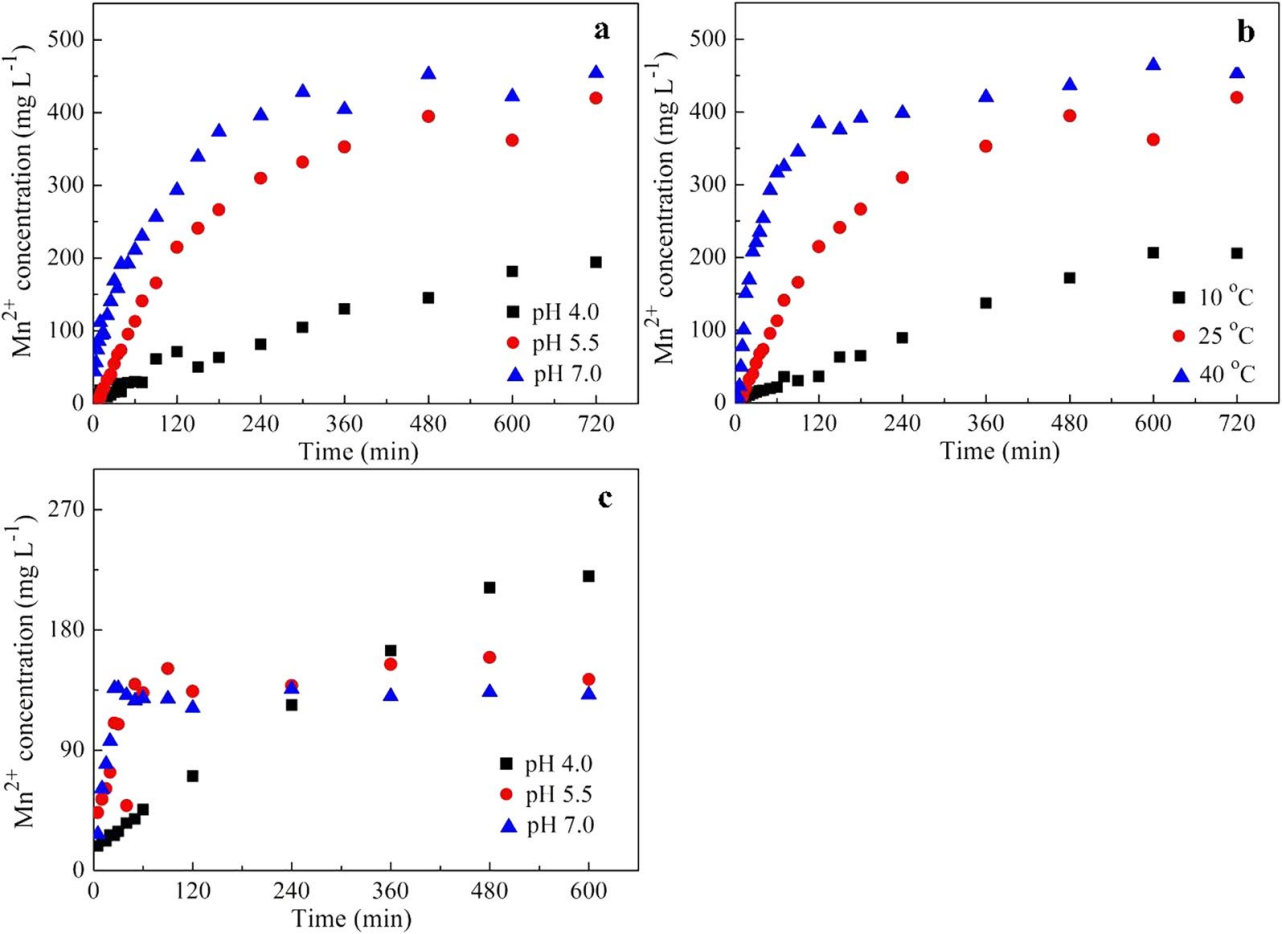
The concentration of released Mn^2+^ in reaction process of 20 mM Fe^2+^ and 1.0 g L^−1^ birnessite under different conditions: **a** pH 4.0–7.0, 25 °C, N_2_; **b** pH 5.5, 10–40 °C, N_2_; **c** pH 4.0–7.0, 25 °C, air

The influence of pH, temperature, and air on the release rate of Mn^2+^ was further studied in the oxidation process of Fe^2+^ of 20 mmol L^−1^ by birnessite of 1.0 g L^−1^. As shown in Fig. 11a, Mn^2+^ release rate increased with an increase of pH in reaction system, suggesting redox rate increasing with an increase in OH^−^ concentration. High pH might also decrease the solubility of Mn^2+^. Therefore, the dissolving rate is not controlled by the transport of reduced species away from the surface, that is to say, Mn^2+^ release into solution is not the rate-determining step [42], which agreed with the results and analysis for the reaction systems with Fe^2+^ of different concentrations. Usually, the oxidation activity of manganese oxide is enhanced with increasing H^+^ concentration [41, 42]. However, increasing pH enriched surface negative charge amount, consequently the adsorption capacity increased [44, 45]), and high pH thermodynamically accelerated redox reaction of Fe^2+^ and manganese oxides [20, 23].

Reaction rate increased with an increase in temperature (Fig. 11b), suggesting a corresponding increase in oxidation activity of birnessite [20]. Another possibility might be attributed to the increase of adsorption capacity as reaction temperature increased. Enhancement of adsorption capacity of birnessite at higher temperatures may be ascribed to the enlargement of pore size and/or activation of the adsorbent surface (Han et al., 2006). When air was bubbled into the aqueous system, compared with the reaction under anoxic condition, reaction rate was increased (Fig. 11a, c) due to the participation of oxygen. The amount of released Mn^2+^ increased within 12 h because of the incomplete redox as shown in Fig. 2. It was also noted that the release rate of Mn^2+^ increased with increasing pH within 4 h, and then it would keep increasing and stable when pH values were controlled at 4.0, 5.5 and 7.0, respectively (Fig. 11c). The redox reaction between birnessite and ferrous ions might be intensified in the late stage likely owing to the higher oxidation activity of birnessite at lower pH. These results further indicated that the presence of oxygen facilitated the chemical stability of birnessite in the initial stage, because Mn(II/III) would be oxidized to Mn(IV) oxide in air atmosphere (Schippers et al., 2005; [28, 41].

**Fig. 12:**
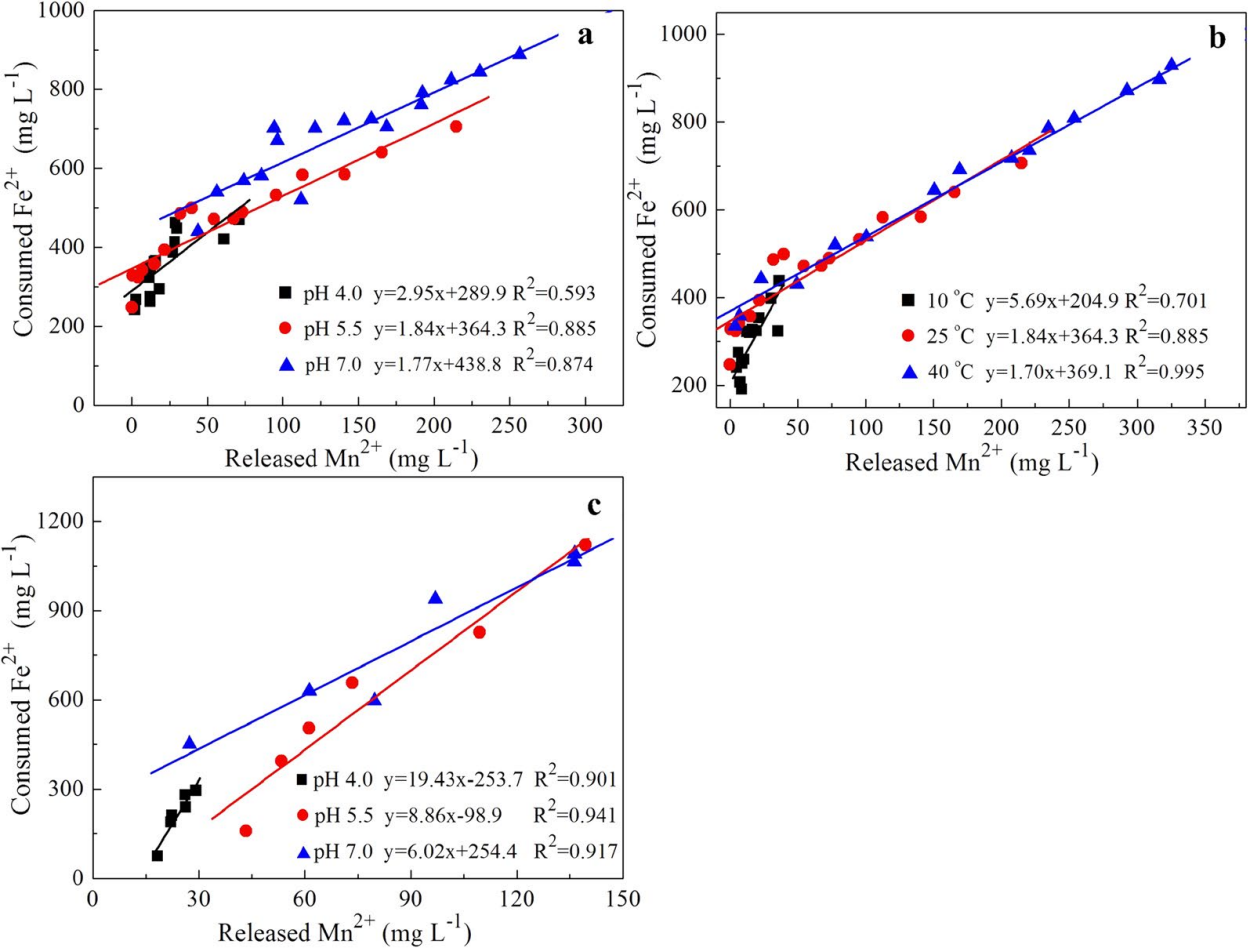
The relationship of consumed Fe^2+^ and released Mn^2+^ concentration in the initial reaction stage of 20 mM Fe^2+^ and 1.0 g L^−1^ birnessite under different conditions: **a** pH 4.0–7.0, 25 °C, N_2_; **b** pH 5.5, 10–40 °C, N_2_; **c** pH 4.0–7.0, 25 °C, air

The redox rate was further analyzed and confirmed by comparing the relationship of consumed Fe^2+^ and released Mn^2+^ concentration in the initial stage. As discussed above, ΔFe/ΔMn slope demonstrates the chemical stability and oxidation capacity of birnessite. From Eq. (1), ΔFe/ΔMn slope of about 1.86 (mass ratio) suggested the balance of adsorption/oxidation of Fe^2+^ and the reduction/release of Mn^2+^, and higher slope of ΔFe/ΔMn indicates higher adsorption/oxidation rate of Fe^2+^ and higher chemical stability of birnessite. As shown in Fig. 12, this slope increased with an increase in pH and a decrease of temperature. These results further revealed that properly increasing alkalinity favored the adsorption and oxidation of Fe^2+^ on the surface of birnessite, resulting in the increase of the release Mn^2+^ concentration. Therefore, ΔFe/ΔMn slope decreased with an increase in pH of reaction system. The lowest slope of 1.70 and the greatest slope of 5.69 were obtained at 10 and 40 °C, respectively. High temperature and proper high pH accelerated the redox reaction and corresponding Mn^2+^ dissolving, and adsorption might be the major reaction at lower temperature.

It was noted that this slope significantly increased from 2.95, 1.84, and 1.77 to 19.43, 8.86, and 6.02 in the presence of air when pH was controlled at 4.0, 5.5, and 7.0, respectively (Fig. 12c). These results indicated the rapid oxidation/adsorption of Fe^2+^ and slow reduction/dissolution of birnessite. Other characterization results indicated that the decrease rate of Fe^2+^ concentration significantly improved (Fig. 5b), and birnessite could be determined after 18 days (Figs. 7b, 4d), and the concentration of released Mn^2+^ dramatically decreased after bubbling air. High pH accelerated the oxidation of Fe^2+^ by birnessite through surface adsorption, and improved the chemical stability, and these factors affected the redox rate at the same time. Combining with these results, it could be safely concluded that the presence of air (oxygen) facilitated the fast oxidation of Fe^2+^ and the improvement of chemical stability of birnessite. These results about the relationship between the oxidized Fe^2+^ and released Mn^2+^ concentration were consistent with the change trend of released Mn^2+^ concentration during the reaction process. Temperature, pH, and oxygen significantly affected the adsorption and oxidation rate with different reaction mechanism.

## Conclusions

The stimulated redox behaviors of ferrous ions and birnessite were studied, and the influence of Fe^3+^, Mn^2+^, temperature, and the presence of air on the transformation process of ferric oxides was investigated with pH 4-7. A mixture of goethite and lepidocrocite was formed with the redox reaction of ferrous ion and birnessite, and lepidocrocite transformed into goethite at the final stage. The transformation from amorphous ferric (hydr)oxide to lepidocrocite and goethite was accelerated by adding Fe^2+^ and Mn^2+^ into Fe_2_(SO_4_)_3_ aqueous solution at pH 5.5, and Fe^2+^ showed better catalytic activity compared with Mn^2+^. A mixed phase of goethite and lepidocrocite was formed within a day. The presence of air (oxygen) improved the chemical stability of birnessite, and significantly accelerated the oxidation rate of ferrous ions. In the oxidation reaction of ferrous ions by oxygen in air, low and high pH values facilitated the formation of goethite and lepidocrocite, respectively. Amorphous ferric (hydr)oxide was formed during the initial stage, and a mixture of poorly crystallized goethite and lepidocrocite was formed after 7 days when pH was controlled at 5.5. A mixture of lepidocrocite and goethite was formed, and the latter was the main product with high crystallinity at pH 7.0. The redox rate was indicated by the disappearing rate of Fe^2+^ and the appearing rate of Mn^2+^ in reaction system. The oxidation rate increased with an increase in the initial concentration of Fe^2+^, pH, and temperature, and the rate was controlled by the redox reaction of adsorbed Fe^2+^ with birnessite, and accelerated in the presence of air.
